# Effect of comprehensive initial training on the variability of left ventricular measures using fast-SENC cardiac magnetic resonance imaging

**DOI:** 10.1038/s41598-019-48685-1

**Published:** 2019-08-21

**Authors:** Tomas Lapinskas, Hanane Hireche-Chikaoui, Victoria Zieschang, Jennifer Erley, Christian Stehning, Rolf Gebker, Sorin Giusca, Grigorios Korosoglou, Remigijus Zaliunas, Sören Jan Backhaus, Andreas Schuster, Burkert Pieske, Sebastian Kelle

**Affiliations:** 1 Department of Internal Medicine/Cardiology, German Heart Center Berlin, Berlin, Germany; 2 Department of Cardiology, Medical Academy, Lithuanian University of Health Sciences, Kaunas, Lithuania; 3 Philips Healthcare, Hamburg, Germany; 4 Department of Cardiology and Vascular Medicine, GRN Hospital Weinheim, Weinheim, Germany; 5 Department of Cardiology and Pneumology, University Medical Center, Georg-August University, Göttingen, Germany; 6 Department of Internal Medicine/Cardiology, Charité Campus Virchow Clinic, Berlin, Germany; 7 DZHK (German Centre for Cardiovascular Research), Partner Site Berlin, Berlin, Germany; 8 DZHK (German Centre for Cardiovascular Research), Partner Site Göttingen, Göttingen, Germany; 9 Department of Cardiology, Royal North Shore Hospital, The Kolling Institute, Northern Clinical School, University of Sydney, Sydney, Australia

**Keywords:** Cardiology, Diagnostic markers

## Abstract

Cardiac magnetic resonance (CMR) is becoming the imaging modality of choice in multicenter studies where highly reproducible measurements are necessary. The purpose of this study was to assess the effect of comprehensive initial training on reproducibility of quantitative left ventricular (LV) parameters estimated using strain-encoded (SENC) imaging. Thirty participants (10 patients with heart failure (HF) and preserved LV ejection fraction (HFpEF), 10 patients with HF and reduced LV ejection fraction (HFrEF) and 10 healthy volunteers) were examined using fast-SENC imaging. Four observers with different experience in non-invasive cardiac imaging completed comprehensive initial training course and were invited to perform CMR data analysis. To assess agreement between observers, LV volumes, mass, ejection fraction (LVEF), global longitudinal strain (GLS) and global circumferential strain (GCS) were estimated using dedicated software (MyoStrain, USA). To test intraobserver agreement data analysis was repeated after 4 weeks. SENC imaging and analysis were fast and were completed in less than 5 minutes. LV end-diastolic volume index (LVEDVi), LVEF and strain were significantly lower in HFpEF patients than in healthy volunteers (p = 0.019 for LVEDVi; p = 0.023 for LVEF; p = 0.004 for GLS and p < 0.001 for GCS). All LV functional parameters were further reduced in HFrEF. Excellent interobserver agreement was found for all LV parameters independently of the level of experience. The reproducibility of LV mass was lower, especially at the intraobserver level (ICC 0.91; 95% CI 0.74–0.96). LV volumetric and functional parameters derived using fast-SENC imaging, are highly reproducible. The appropriate initial training is relevant and allows to achieve highest concordance in fast-SENC measurements.

## Introduction

In addition to clinical signs and symptoms, a detailed assessment of structural and functional cardiac parameters is considered to be essential and provides important diagnostic information in patients with heart failure (HF)^1,2^. Over the past decade cardiac magnetic resonance (CMR) has evolved into the reference standard to assess cardiac anatomy and function^3,4^. Because of its excellent endocardial border definition, cine CMR imaging is the accepted gold standard for quantification of ventricular volumes, mass and ejection fraction^5,6^. While important achievements in CMR techniques have reduced total scan time, quantitative volumetric analysis has not changed significantly and requires time and human resources^7,8^.

Growing evidence suggests that conventional functional parameters such as left ventricular ejection fraction (LVEF) may not be sensitive enough to detect subtle changes in ventricular function^9^. Tissue-tracking techniques have enabled the non-invasive assessment of myocardial deformation and it appears that myocardial strain might be a more robust marker of the failing myocardium^10–12^. Strain-encoded magnetic resonance (SENC) imaging was developed on the concepts of myocardial tagging and first described in 2001^13^. In line with other tissue tracking techniques, SENC provides quantitative information about myocardial mechanics and has been validated and applied in multiple experimental and clinical settings^14–16^. Fast-SENC technique is a real-time version of SENC that has shortened the scan duration to a single heartbeat^17^. The absence of contrast agent and free breathing during data acquisition are important advantages that make the technique desirable in daily routine. Moreover, recent achievements in fast-SENC data analysis tools have enabled quantification of conventional left ventricular (LV) volumetric and functional parameters with excellent accuracy and minimal educational efforts^18^.

More and more clinical decision making relies on CMR derived data^19–21^; hence, interobserver and intraobserver variability may become an important source of bias. Although recommendations exist for CMR acquisition^22^ and data post-processing^23,24^, the lack of proper initial training of the observers may lead to significant measurement variance which becomes more apparent in multicenter studies. Indeed, appropriate education^25,26^ as well as repeated measurements^27^ might improve interobserver reproducibility for volumetric and functional measures of the left ventricle.

We set up this study to investigate the effect of comprehensive initial training on reproducibility of LV volumes, mass, ejection fraction and strain derived using fast-SENC imaging. The main hypothesis was that appropriate initial training has an important impact in terms of cardiac imaging of the readers on the concordance of measurements.

## Materials and Methods

### Study population

The study population comprised patients with heart failure and preserved LVEF (HFpEF, n = 10), reduced LVEF (HFrEF, n = 10) and healthy volunteers (n = 10). The study complies with the Declaration of Helsinki and was approved by the ethics committee board of the Charité-Universitätsmedizin Berlin. All participants provided written informed consent before entering the study.

### CMR acquisition

All CMR studies were performed on a 1.5 T MRI scanner (Achieva, Philips Healthcare, Best, The Netherlands) using a five-element phased array cardiac coil in supine position.

A previously described^17^ real-time free breathing SENC imaging technique (Myocardial Solutions, Inc., Morrisville, North Carolina, USA) based on the acquisition of two images with different frequency modulation was employed. Images were taken in three different LV long-axis (two-, three- and four-chamber) and three LV short-axis (at basal, mid-ventricular and apical level) views. The slices of different LV short-axis levels were identified as follows: basal level slice was considered and used for quantitative analysis if complete LV myocardium was visible throughout the entire cardiac cycle; mid-ventricular level slice was selected at the level of both papillary muscles; and apical level slice was considered if blood pool was still visible throughout the entire cardiac cycle (no obliteration of the LV cavity at end-systolic phase). Typical fast-SENC parameters were as follows: field-of-view = 256 × 256 mm^2^, slice thickness = 10 mm, voxel size = 4.0 × 4.0 × 10 mm^3^, single-shot spiral readout (3 interleaves) with acquisition time (TA) = 10 ms, flip angle = 30°, effective echo time (TE) = 0.7 ms, repetition time (TR) = 12 ms, temporal resolution = 36 ms, typical number of acquired phases = 22, spectrally selective fat suppression (SPIR), total acquisition time per slice < 1 s.

### Study observers

The four observers with different knowledge and experience in CMR imaging were invited to perform analysis of acquired CMR data: (1) CMR expert (TL) (level 3 certified, performing routine clinical CMR scanning and data post-processing for >5 years); (2) CMR beginner (VZ) (with basic knowledge and <3 months of experience in CMR imaging); (3) Echo expert (HHC) (level 3 certified, performing advanced echocardiography studies including speckle tracking echocardiography in high-volume cardiovascular unit) and (4) Non-cardiac technician (JE) (fully-trained radiographer without any experience in data post-processing).

### Training protocol

**Figure 1:**
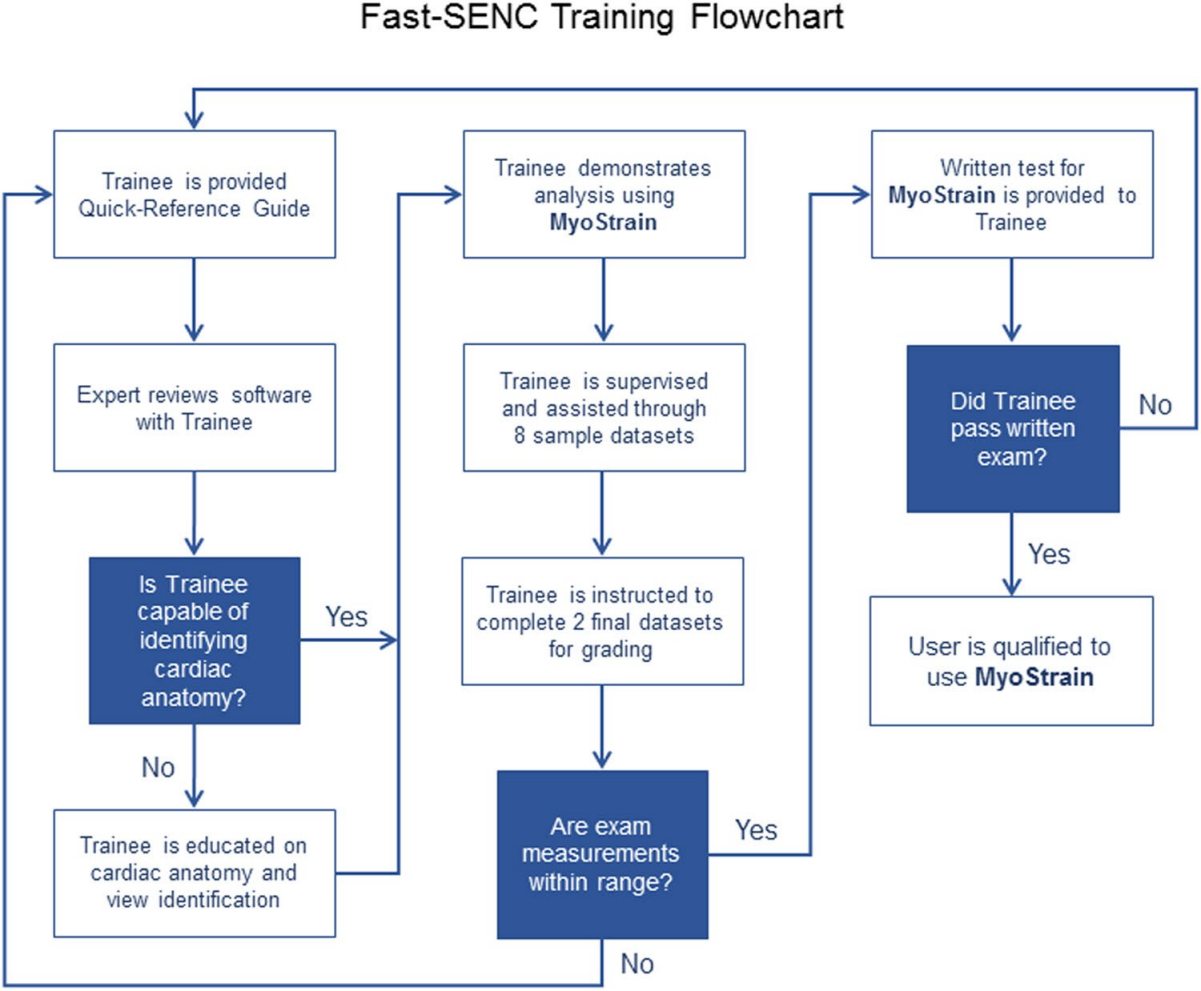
Fast-SENC training flowchart. Before starting the analysis of CMR data, observers acquired similar comprehensive expert-guided training. The training consisted of a 2 to 4-hour training course designed to provide the skills necessary to correctly use the software. After successful completion of the training course and examination observers were allowed to start study data analysis.

Before starting the CMR data analysis, all observers were trained similarly by a representative of the software company with an emphasis on possible sources of error. A Quick-Reference Guide was given to each trainee before starting the training. The training consisted of a 2 to 4-hour training course designed to provide observers with the skills necessary to correctly use the dedicated MyoStrain software (Fig. 1). A set of 8 cases was used during the training including different cardiac conditions (healthy, cardiomyopathies, ischemic heart disease) and possible image quality issues (suboptimal field-of-view, patient movement or image artifacts) which may arise when using the software. Each trainee had a hands-on analysis session in a blinded manner with personal feedback from the expert. Observers were instructed not to analyze if they thought image quality was inadequate. After completion of the 8 cases, 2 additional datasets were provided and analyzed independently from the expert. The analyses were collected and reviewed by the training site to ensure that measurements were performed correctly. Estimates collected from these analyses had to fall within acceptable ranges. A written test was also mandatory and comprised 50 questions covering cardiac anatomy, view identification and image quality as well as identification of systolic and diastolic cardiac phases. A score of 80% or above on the written exam was considered “passed”. After completion of the training course and positive feedback from the training site, observers were allowed to start study data analysis.

### Image analysis

**Figure 2:**
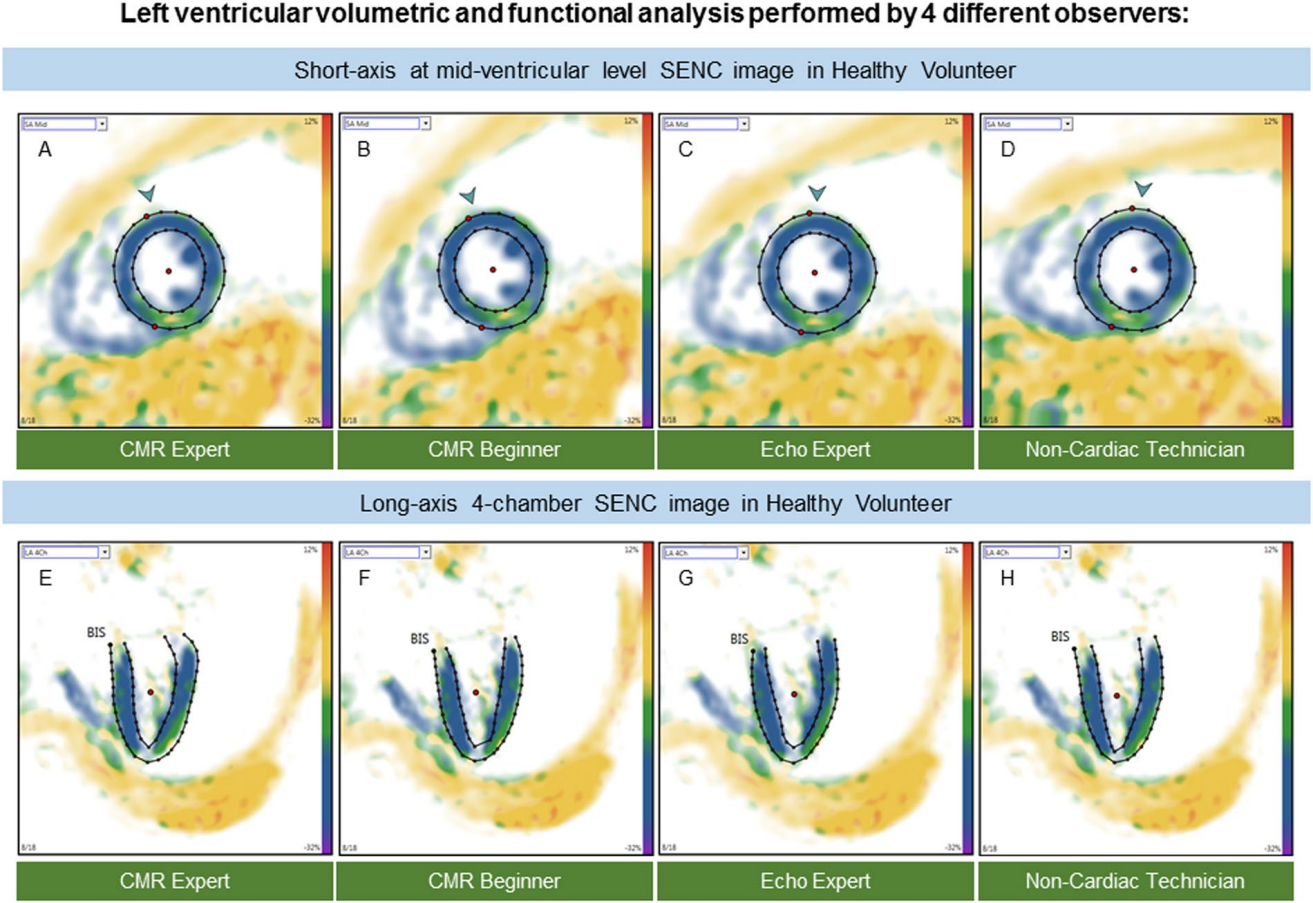
Example of fast-SENC images acquired in a healthy volunteer and uploaded into a dedicated MyoStrain software. CMR images were derived in three long-axis and three short-axis views. Endocardial and epicardial borders were traced at end-diastolic and end-systolic cardiac phases by four observers: CMR expert (**A**,**E**) CMR beginner (**B**,**F**) echocardiography expert (**C**,**G**) and non-cardiac technician (**D**,**H**). CMR = cardiac magnetic resonance; SENC = strain-encoded imaging.

**Figure 3:**
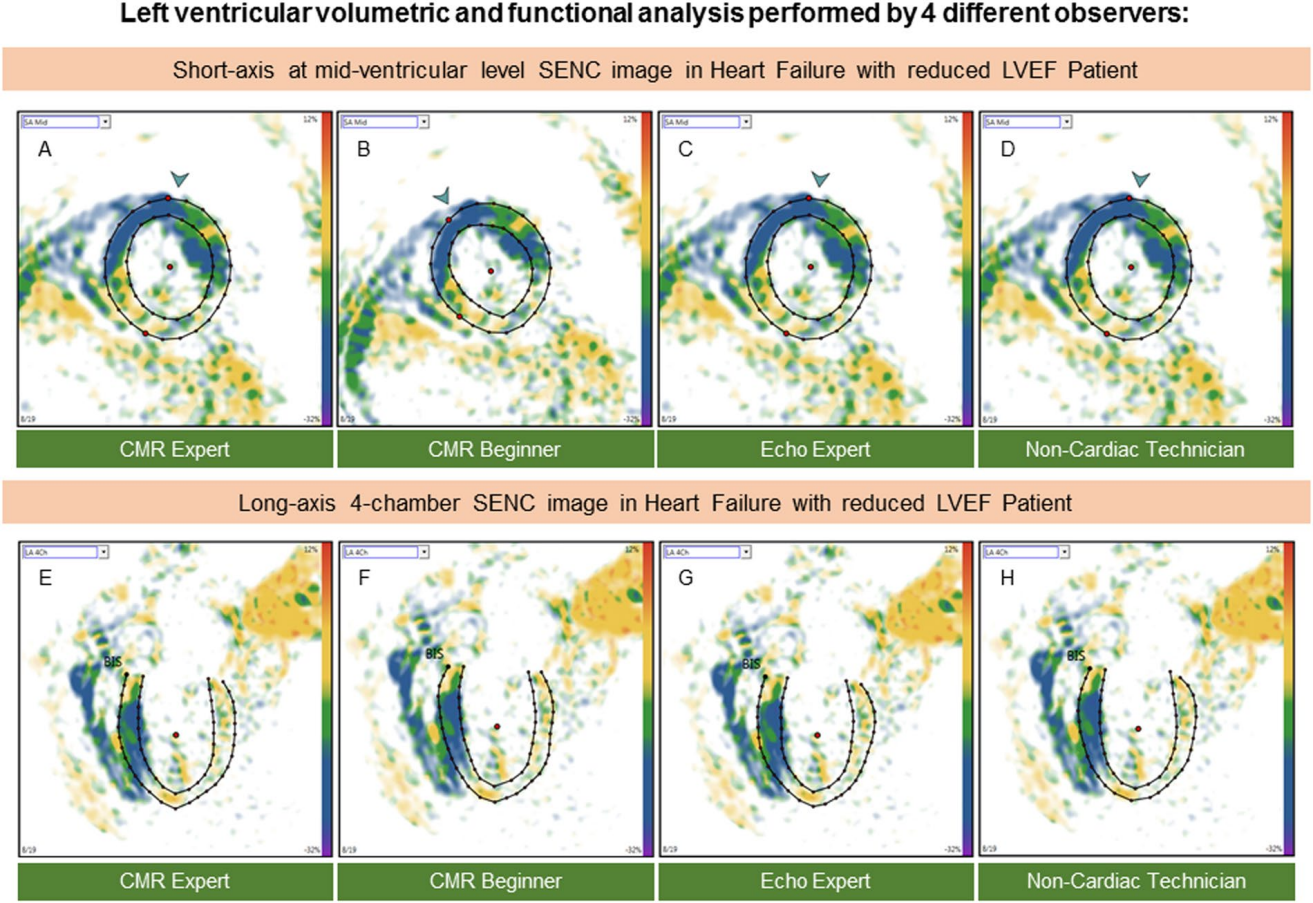
Example of fast-SENC images acquired in HFrEF patients and uploaded into a dedicated MyoStrain software. Endocardial and epicardial borders were traced at end-diastolic and end-systolic cardiac phases by four observers: CMR expert (**A**,**E**) CMR beginner (**B**,**F**) echocardiography expert (**C**,**G**) and non-cardiac technician (**D**,**H**). CMR = cardiac magnetic resonance; SENC = strain-encoded imaging; HFrEF = heart failure with reduced left ventricular ejection fraction.

All fast-SENC images were uploaded from the MRI scanner and analyzed using dedicated MyoStrain, version 4.2 software (Morrisville, NC, USA). LV end-diastolic (LVEDV), LV end-systolic volumes (LVESV) and LV mass (LVM) were quantified using manual planimetry of the endocardial and epicardial surface from three long-axis fast-SENC images and LVEF was calculated (Figs 2 and 3). The quality of the contouring was evaluated by visually comparing the tracking process with the underlying myocardial motion. Papillary muscles were considered part of the blood pool. LV volumes and mass were adjusted to body surface area. The LV longitudinal and circumferential strain was extracted from three LV short-axis and three LV long-axis fast-SENC images, respectively. The global strain values were calculated by averaging measurements obtained from 16 segments for global longitudinal strain (GLS) and 17 segments for global circumferential strain (GCS).

### Statistical analysis

Data were analyzed using IBM SPSS Statistics, version 21.0 software (SPSS Inc., Chicago, IL, USA) for Windows. Continuous variables were expressed as mean ± standard deviation. The distribution of continuous variables was assessed using the Shapiro-Wilk test and comparisons between groups were performed with the 2-sample *t* test and the Mann-Whitney U test where appropriate. Interobserver and intraobserver variability was assessed by intraclass correlation (ICC) (2-way mixed model, absolute agreement between single measurements) and Bland-Altman analysis^28^. Agreement was considered excellent for ICC >0.74, good for ICC 0.60–0.74, fair for ICC 0.40–0.59, and poor for ICC <0.40^29^. A p value of <0.05 was considered statistically significant.

### Ethics approval and consent to participate

The local ethics committee (Charité-Universitätsmedizin Berlin) approved the research and consent was obtained for all study participants.

## Results

All study participants were able to complete the entire study protocol. SENC imaging and analysis was fast with a 15 second scan time and a 3 to 5 minute post-processing time for complete quantitative assessment including LV volumes, mass, ejection fraction and global longitudinal and circumferential strain.

There was no significant difference in LVESV and LVM indices between healthy volunteers and HFpEF patients (p = 0.579 for LVESVi and p = 0.315 for LVMi), while LVEDVi, LVEF and strain values were significantly lower in HFpEF (87.80 ± 7.65 ml/m^2^ vs. 75.20 ± 11.66 ml/m^2^, p = 0.019 for LVEDVi; 60.41 ± 5.57% vs. 55.96 ± 3.40%, p = 0.023 for LVEF; −20.45 ± 1.46% vs. −18.92 ± 0.84%, p = 0.004 for GLS and −21.25 ± 1.19% vs. −17.35 ± 1.89%, p < 0.001 for GCS). All LV functional parameters were further reduced in HFrEF patients compared with healthy volunteers or HFpEF. LV volumes and mass were significantly larger in HFrEF than in other study subjects. Table 1 demonstrates fast-SENC derived parameters in the study population.

**Table 1:** Comparison of LV volumes, mass, ejection fraction and strain parameters among healthy volunteers and patients with heart failure with preserved (HFpEF) and reduced LV ejection fraction (HFrEF).

Parameter	Volunteers (n = 10)	HFpEF (n = 10)	HFrEF (n = 10)	P value		
				Volunteers vs. HFpEF	Volunteers vs. HFrEF	HFpEF vs. HFrEF
LVEDVi (ml/m^2^)	87.80 ± 7.65	75.20 ± 11.66	133.74 ± 22.00	0.019	<0.001	<0.001
LVESVi (ml/m^2^)	34.71 ± 5.35	33.42 ± 7.38	100.31 ± 26.51	0.579	<0.001	<0.001
LVEF (%)	60.41 ± 5.57	55.96 ± 3.40	25.64 ± 10.45	0.023	<0.001	<0.001
LVMi (g/m^2^)	55.36 ± 7.35	59.89 ± 9.25	88.60 ± 17.22	0.315	<0.001	<0.001
GLS (%)	−20.45 ± 1.46	−18.92 ± 0.84	−10.92 ± 4.33	0.004	<0.001	<0.001
GCS (%)	−21.25 ± 1.19	−17.35 ± 1.89	−11.89 ± 3.07	<0.001	<0.001	<0.001

Results are reported as mean ± standard deviation. LV = left ventricular; LVEDVi – left ventricular end-diastolic volume index; LVESVi = left ventricular end-systolic volume index; LVEF = left ventricular ejection fraction; LVMi = left ventricular mass index; GLS = global longitudinal strain; GCS = global circumferential strain; HFpEF = heart failure with preserved ejection fraction; HFrEF = heart failure with reduced ejection fraction.

**Figure 4:**
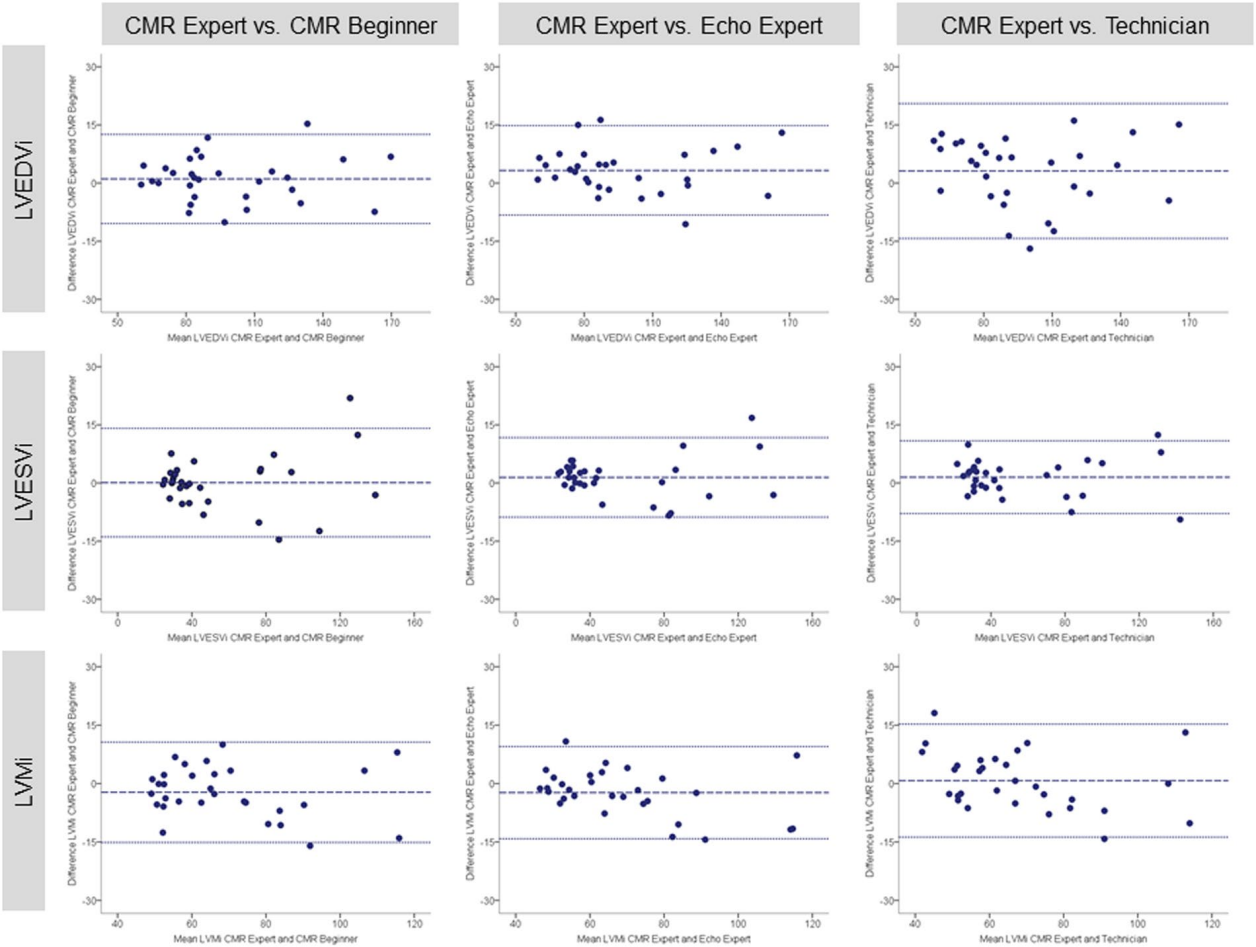
Bland-Altman plots with limits of agreement (1.96 standard deviations) demonstrate the interobserver agreement of fast-SENC for LVEDVi, LVESVi and LVMi. The middle-dashed line is the mean of difference of measures. The upper and lower dotted lines are ±1.96 standard deviation. SENC = strain-encoded imaging; LVEDVi = left ventricular end-diastolic volume index; LVESVi = left ventricular end-systolic volume index; LVMi = left ventricular mass index.

**Figure 5:**
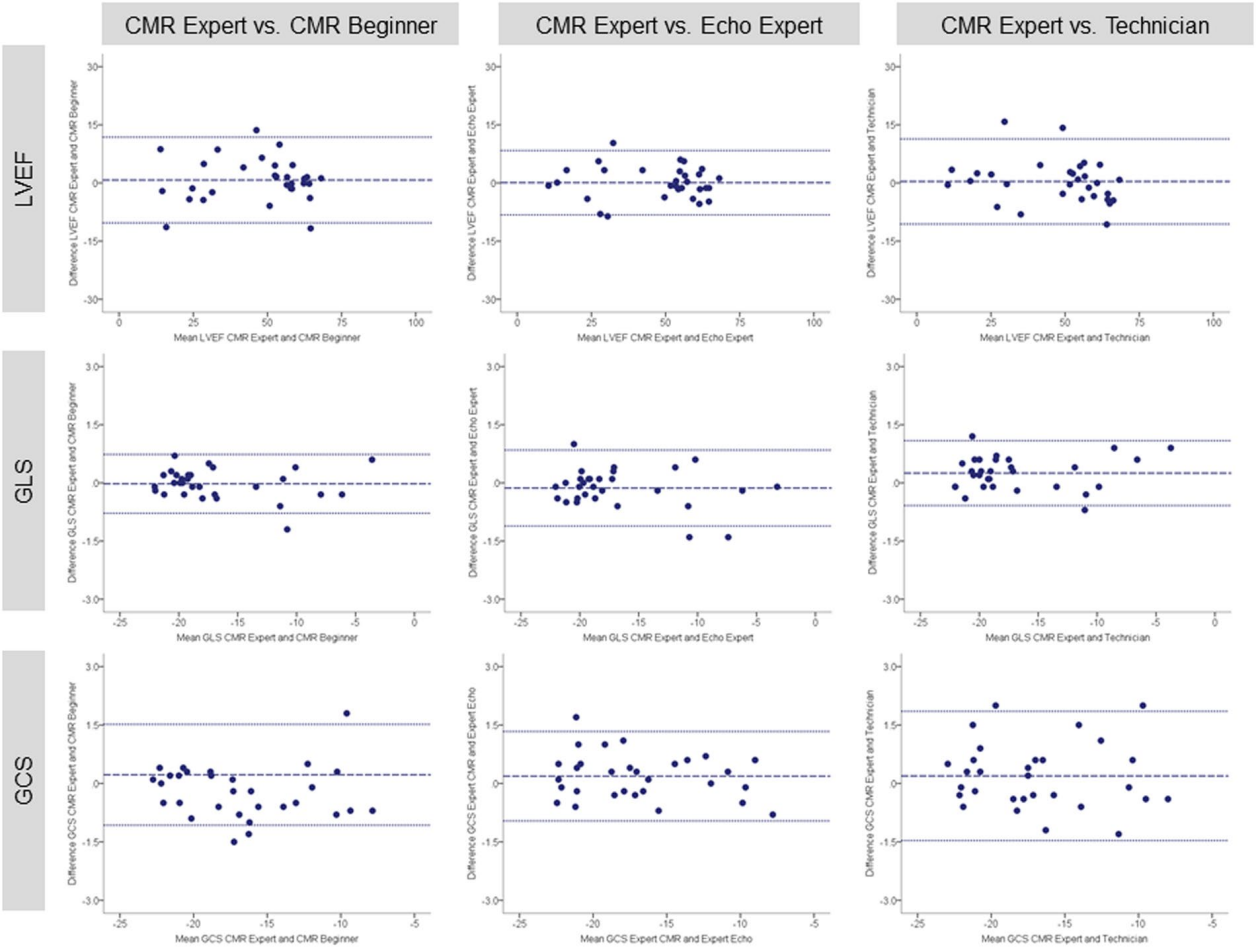
Bland-Altman plots with limits of agreement (1.96 standard deviations) demonstrate the interobserver agreement of fast-SENC for LVEF, GLS and GCS. LVEF = left ventricular ejection fraction; GLS = global longitudinal strain; GCS = global circumferential strain.

**Figure 6:**
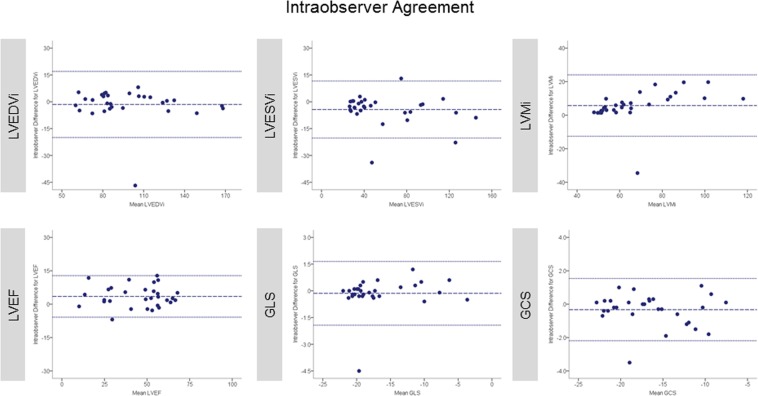
Bland-Altman plots with limits of agreement show the intraobserver agreement of fast-SENC for LVEDVi, LVESVi, LVMi, LVEF, GLS and GCS. The middle-dashed line is the mean of difference of measures. The upper and lower dotted lines are ±1.96 standard deviation. SENC = strain-encoded imaging; LVEDVi = left ventricular end-diastolic volume index; LVESVi = left ventricular end-systolic volume index; LVMi = left ventricular mass index; LVEF = left ventricular ejection fraction; GLS = global longitudinal strain; GCS = global circumferential strain.

Excellent interobserver reproducibility was found for volumetric and functional LV parameters independently of the previous reader’s experience. The least reproducible measure was LVMi with lowest agreement at intraobserver level (ICC 0.91; 95% confidence interval 0.74–0.96) (intraobserver analysis was performed by CMR beginner). Tables 2 and 3 summarize values for mean difference ± SD, limit of agreement and ICC between study observers for LV volumes, mass and function. Correspondingly, Bland-Altman plots for LVEF and strain are displayed in Figs 4 and 5. Bland-Altman plots for intraobserver reproducibility are depicted in Fig. 6.

**Table 2:** Bland-Altman analysis and ICC of pairwise comparison between study observers for LV volumes, mass, ejection fraction and strain parameters (analysis of entire study population, n = 30).

Parameter	Mean difference ± SD	Limits of agreement	ICC (95% CI)
	CMR Expert vs. CMR Beginner		
LVEDVi (ml/m^2^)	1.07 ± 5.86	−10.42 to 12.56	0.99 (0.98 to 1.00)
LVESVi (ml/m^2^)	0.10 ± 7.14	−13.89 to 14.09	0.99 (0.98 to 1.00)
LVMi (g/m^2^)	−2.24 ± 6.57	−15.13 to 10.64	0.97 (0.93 to 0.99)
LVEF (%)	0.76 ± 5.64	−10.30 to 11.82	0.97 (0.94 to 0.99)
GLS (%)	−0.02 ± 0.39	−0.78 to 0.73	1.00 (1.00 to 1.00)
GCS (%)	0.22 ± 0.66	−1.07 to 1.52	0.99 (0.99 to 1.00)
	**CMR Expert vs**. **Echo Expert**		
LVEDVi (ml/m^2^)	3.29 ± 5.89	−8.25 to 14.83	0.99 (0.96 to 1.00)
LVESVi (ml/m^2^)	1.45 ± 5.24	−8.81 to 11.72	0.99 (0.99 to 1.00)
LVMi (g/m^2^)	−2.32 ± 6.04	−14.16 to 9.51	0.98 (0.94 to 0.99)
LVEF (%)	0.07 ± 4.23	−8.22 to 8.36	0.99 (0.97 to 0.99)
GLS (%)	−0.13 ± 0.50	−1.11 to 0.85	1.00 (1.00 to 1.00)
GCS (%)	0.19 ± 0.59	−0.96 to 1.33	1.00 (0.99 to 1.00)
	**CMR Expert vs**. **Non**-**Cardiac Technician**		
LVEDVi (ml/m^2^)	3.12 ± 8.87	−14.27 to 20.51	0.98 (0.95 to 0.99)
LVESVi (ml/m^2^)	1.51 ± 4.79	−7.88 to 10.90	1.00 (0.99 to 1.00)
LVMi (g/m^2^)	0.75 ± 7.40	−13.76 to 15.25	0.97 (0.93 to 0.98)
LVEF (%)	0.38 ± 5.60	−10.60 to 11.35	0.98 (0.95 to 0.99)
GLS (%)	0.25 ± 0.43	−0.58 to 1.09	1.00 (0.99 to 1.00)
GCS (%)	0.19 ± 0.85	−1.47 to 1.85	0.99 (0.98 to 1.00)

Results are reported as mean ± standard deviation. ICC = intraclass correlation coefficient; CI = confidence interval; CMR = cardiac magnetic resonance. Other abbreviations as in Table 1.

**Table 3:** Bland-Altman analysis and ICC of pairwise comparison to assess intraobserver agreement for LV volumes, mass, ejection fraction and strain parameters (analysis of entire study population, n = 30).

Parameter	First measurement	Second measurement	Mean difference	Limits of agreement	ICC (95% CI)
LVEDVi (ml/m^2^)	97.84 ± 29.26	99.36 ± 30.31	−1.52 ± 9.42	−19.98 to 16.95	0.98 (0.95 to 0.99)
LVESVi (ml/m^2^)	56.05 ± 34.16	60.38 ± 35.90	−4.33 ± 8.13	−20.27 to 11.60	0.98 (0.95 to 0.99)
LVMi (g/m^2^)	70.19 ± 20.20	64.45 ± 16.45	5.75 ± 9.34	−12.57 to 24.06	0.91 (0.74 to 0.96)
LVEF (%)	46.58 ± 17.02	43.10 ± 17.01	3.48 ± 4.76	−5.85 to 12.81	0.97 (0.88 to 0.99)
GLS (%)	−16.74 ± 5.03	−16.60 ± 4.87	−0.14 ± 9.11	−1.93 to 1.65	0.99 (0.98 to 1.00)
GCS (%)	−16.61 ± 4.47	−16.28 ± 4.58	−0.33 ± 0.95	−2.19 to 1.54	0.99 (0.97 to 0.99)

Results are reported as mean ± standard deviation. Abbreviations as in Table 1.

## Discussion

The current study was designed to test whether comprehensive initial training has more relevant impact on the reproducibility of LV volumetric and functional parameters estimated using fast-SENC technique than observers experience. Our data analysis demonstrated that:

- LV volumetric and functional parameters can be precisely derived from fast-SENC images in a single short data analysis session.
- Appropriate initial training is important and has impact on the concordance in measurements among observers independently of their previous experience.
- Excellent interobserver and intraobserver agreement is present for all quantitative LV parameters, especially for GLS and GCS, whereas measures of LV mass appear less robust.

The assessment of LV function is probably the most important part of every cardiac imaging study. The majority of clinical decision making algorithms largely rely on quantitative variables such as LVEF^30–32^. It has been shown that this single measure is critical for diagnosis of HF and selection of optimal medical or device therapy^33^. However, there is evidence that advanced measures of myocardial performance, such as strain or torsion, are better predictors of outcome than LVEF or wall motion score index^34,35^. Tissue-tracking techniques such as speckle tracking echocardiography, CMR tagging, displacement encoded with stimulated echoes (DENSE) imaging or feature tracking appear very promising and have shown the ability to detect early changes in myocardial motion^36^. Historically, CMR tagging was the first technique implemented for the analysis of myocardial deformation^37^. However, time-consuming data acquisition and analysis remain important limitations of this standard of reference technique^38,39^.

In 2001 Osman *et al*., proposed a new method for measuring the myocardial strain orthogonal to the imaging plane, called SENC-MRI^13^. The method required the acquisition of two images and allowed straightforward and fast computation of longitudinal strain. Recent achievements in SENC technique have shortened the scan duration to a single heartbeat and eliminated the demand of multiple breath-holds^40^. Fast-SENC was validated against the conventional CMR tagging and excellent correlation between the methods was shown^41,42^. The ability to obtain accurate measurements in a short time is highly desirable, especially in severely ill patients and children. We successfully completed image acquisition and data analysis in less than five minutes, while participants were still in the MRI scanner. Such achievements make the implementation of this technique in the clinical realm very promising.

The reliability and reproducibility of LV functional measures are of great importance for patient management, therapy monitoring and outcome studies^43^. A lower level of variability permits detection of smaller changes and may reduce the necessary sample size for clinical trials^44,45^. Neizel *et al*., evaluated interobserver agreement in healthy volunteers and found very high reproducibility in SENC strain measurements (r = 0.87), which was superior to CMR tagging (0.81)^42^. Hamdan *et al*., reported similar findings in healthy volunteers scanned on a 3.0 T MRI system with ICC between observers and repeated studies ranging from 0.92 to 0.98^46^. Our findings are in line with the results of previous studies. We found excellent interobserver and intraobserver agreement of LV global longitudinal and circumferential strain. Moreover, for the first time we demonstrated excellent interobserver and intraobserver reproducibility of LV volumes and ejection fraction estimated using fast-SENC imaging. As reported previously, the agreement of LV mass measurements was lower, especially at the intraobserver level (ICC 0.91; 95% confidence interval 0.74–0.96).

At present, CMR is becoming the imaging modality of choice in multicentric cardiovascular trials^47^; therefore, inter-institutional agreement of derived measures must be recognized as a relevant source of error. Sample size calculation is an important aspect of study design and depends on the concordance of measurements. Much attention has been attributed to CMR scanning and data analysis guidelines^22–24^. However, little is known on whether knowledge, experience or appropriate initial training has more impact on the precision of measurements. Beerbaum *et al*., investigated the impact of interobserver variance between the institutions for volumetric and flow CMR data. Images were analyzed by experienced readers only. Inter-institutional agreement was assessed before and after a dedicated training course. Interestingly, in patients, on transverse planes, variation coefficient for LV volumes was significantly decreased by training (p < 0.007). For short-axis volumetry training also resulted in narrower limits of agreement. The reproducibility did not improve significantly with training in healthy volunteers. However, the highest variability after training in volunteers was found for LV mass (transverse acquisition: 12–15%, short-axis acquisition: 9–12%)^48^.

In a recent study, Negishi *et al*. evaluated the role of experience in the precision and validity of strain measurements derived using speckle tracking echocardiography. Their study revealed that although the group with the highest level of experience achieved better agreement than those with no experience, the ICC of the inexperienced observers was still very high (0.996 vs. 0.975; p = 0.0002)^49^. To examine the importance of initial training we selected four study observers with different knowledge and experience background but provided comprehensive and expert-guided training. Data analysis demonstrated that interobserver agreement was excellent independently of readers’ expertise. Nevertheless, it should be noted that LV mass measurements were more variable, especially at the intraobserver level (analysis performed by a CMR beginner), confirming that degree of experience might be also important and should not be underestimated. Despite higher variability, the concordance of LV mass measurements is still clinically acceptable (ICC 0.91).

Our very recent study was conducted to evaluate the impact of proper training on the variability of myocardial strain measurements derived using different commercially available CMR feature tracking software packages. Study results demonstrated that dedicated training of the observer significantly improves reproducibility of LV GLS and GCS^50^. Findings of this study are in line with previous studies and confirm that appropriate initial training might be more important to achieve highest concordance in CMR measurements. In the light of inadequate experience in CMR imaging fast-SENC technique would be highly desirable in non-expert CMR centers where precise quantitative LV analysis could be performed rapidly even by unexperienced readers.

## Limitations

Several limitations of the current study should be noted. First, the population of this study was relatively small. Second, it was single center, single vendor, single software and single MRI lab protocol. Third, only fast-SENC imaging was used to investigate the performance of observers without reflection of other CMR tissue tracking techniques such as tagging, DENSE or feature tracking. We also did not compare fast-SENC images acquired by MRI scanners of different vendors. Lastly, we did not assess the variability of measurements before the training of the observers.

## Conclusion

Excellent reproducibility of LV volumetric and functional parameters makes fast-SENC a reliable imaging modality for future studies. Although level of experience is important, it appears that appropriate initial training has much more impact on the agreement of derived measurements. However, larger multicenter studies using MRI scanners and software packages from different vendors are necessary to confirm our findings.

## Data Availability

The datasets used and/or analyzed during the current study are available from the corresponding author on reasonable request.
